# Supplementary material for: A double dissociation between semantic and spatial cognition in visual to default network pathways
Source: eLife. 2025 Jan 22;13:RP94902. doi: 10.7554/eLife.94902 (PMC11753780; doi:10.7554/eLife.94902)
Supplement: Supplementary file 1. [file elife-94902-supp1.docx]

Supplementary File 1. Cluster Information for Neuroimaging Results from Study 1

|  |  |  |  | Coordinates (in mm) | | |  |
| --- | --- | --- | --- | --- | --- | --- | --- |
| Analysis | Hemisphere | Cluster Peak | Z | x | y | z | Cluster Volume |
| Probe: Semantic > Spatial Context | Left | Lateral Occipital Cortex, Inferior Division | 4.78 | -50 | -74 | 4 | 932 |
|  | Right | Lateral Occipital Cortex, Inferior Division | 4.58 | 50 | -68 | 0 | 534 |
|  | Left | Parietal Operculum Cortex | 3.9 | -54 | -34 | 22 | 218 |
| Probe: Spatial Context > Semantic | Right | Occipital Pole | 6.64 | 14 | -92 | 2 | 9931 |
|  | - | Paracingulate Gyrus | 3.82 | 0 | 16 | 52 | 495 |
|  | Left | Frontal Pole | 3.78 | -30 | 60 | 14 | 342 |
|  | Left | Frontal Orbital / Insular Cortex | 4.42 | -30 | 26 | -2 | 236 |
|  | Right | Middle Frontal Gyrus | 3.73 | 32 | 2 | 64 | 218 |
|  | Left | Lateral Occipital Cortex, Superior division | 3.7 | -36 | -60 | 54 | 218 |
| Decision: Semantic > Spatial Context | Left | Lateral Occipital Cortex, Inferior Division | 5.24 | -50 | -74 | 4 | 2438 |
|  | Right | Lateral Occipital Cortex, Inferior Division | 5.32 | 50 | -70 | 2 | 1605 |
|  | Left | Middle / Inferior Frontal Gyrus | 4.6 | -50 | 28 | 24 | 1369 |
|  | Left | Middle Frontal / Precentral Gyrus | 4.57 | -52 | 8 | 40 | 385 |
|  | Left | Intracalcarine Cortex | 3.92 | -6 | -76 | 12 | 363 |
|  | Left | Frontal Pole / Superior Frontal Gyrus | 3.55 | -12 | 40 | 52 | 217 |
|  | Right | Frontal Operculum Cortex | 4.04 | 46 | 18 | -6 | 207 |
| Decision: Spatial Context > Semantic | Right | Occipital Pole | 4.62 | 4 | -94 | -6 | 1543 |
|  | Right | Temporal Occipital Fusiform Cortex | 5.34 | 30 | -46 | -8 | 420 |
|  | Right | Precuneous Cortex | 4.17 | 20 | -58 | 18 | 253 |
|  | Left | Lingual Gyrus | 5.12 | -24 | -46 | -8 | 251 |
| Probe: Spatial Context Mixed > Same | Right | Lateral Occipital Cortex, Superior division | 4.06 | 50 | -70 | 32 | 294 |
|  | Left | Precuneous Cortex | 3.71 | -8 | -70 | 28 | 232 |
| Probe: Spatial Context Same > Mixed | Right | Angular Gyrus | 3.85 | 58 | -50 | 44 | 222 |
| Probe: Semantic Mixed > Same | Left | Lateral Occipital Cortex, Superior division | 3.99 | -36 | -60 | 54 | 610 |
|  | Right | Superior Parietal Lobule | 3.51 | 38 | -54 | 58 | 364 |
|  | Left | Lateral Occipital Cortex, inferior division | 3.36 | -50 | -66 | -18 | 286 |
|  | Right | Inferior Temporal Gyrus, temporooccipital part | 3.45 | 50 | -60 | -22 | 278 |
|  | Right | Lateral Occipital Cortex, Superior division | 3.57 | 40 | -78 | 38 | 265 |
