# Supplementary material for: A double dissociation between semantic and spatial cognition in visual to default network pathways
Source: eLife. 2025 Jan 22;13:RP94902. doi: 10.7554/eLife.94902 (PMC11753780; doi:10.7554/eLife.94902)
Supplement: Supplementary file 2. [file elife-94902-supp2.docx]

Supplementary File 2. Paired t-tests contrasting spatial similarity of participant-level activation with group-level context and semantic pathways and non-pathways

| Individual Task Responses | Group-defined pathways | Non-pathway conjunctions | t | df | Two-sided p |
| --- | --- | --- | --- | --- | --- |
| Context Decision | Context Pathway (Context Probe and Context Decision) | Context Probe and Semantic Decision | 76.25 | 190 | 2E-144 |
| Context Decision | Context Pathway (Context Probe and Context Decision) | Semantic Probe and Context Decision | 70.89 | 190 | 1.3E-138 |
| Semantic Decision | Semantic Pathway (Semantic Probe and Semantic Decision) | Context Probe and Semantic Decision | 17.55 | 190 | 1.22E-41 |
| Semantic Decision | Semantic Pathway (Semantic Probe and Semantic Decision) | Semantic Probe and Context Decision | 52.95 | 190 | 1E-115 |
| Context Probe | Context Pathway (Context Probe and Context Decision) | Context Probe and Semantic Decision | 2.36 | 190 | 0.019292 |
| Context Probe | Context Pathway (Context Probe and Context Decision) | Semantic Probe and Context Decision | 31.11 | 190 | 1.74E-76 |
| Semantic Probe | Semantic Pathway (Semantic Probe and Semantic Decision) | Context Probe and Semantic Decision | 14.2 | 190 | 1.14E-31 |
| Semantic Probe | Semantic Pathway (Semantic Probe and Semantic Decision) | Semantic Probe and Context Decision | 18.01 | 190 | 5.68E-43 |

Note. T values represent contrasts between the correlations of individual task responses and group-defined pathways versus these same responses correlated with non-pathway conjunctions (pathway > non-pathway)
